# Building global research equity through lung organoids: a prerequisite for the next respiratory pandemic

**DOI:** 10.3389/fmed.2025.1733234

**Published:** 2026-01-12

**Authors:** Muhammad Kamal Hossain, Hyung-Ryong Kim

**Affiliations:** 1 The Organoid Society, Hall 1 of Jeonbuk National University, Dental College, Jeonju-si, South Korea; 2 Organoid Laboratory, Department of Pharmacology, School of Dentistry, Jeonbuk National University, Jeonju, Republic of Korea; 3 Non-Clinical Evaluation Center (NCEC), Biomedical Research Institute, Jeonbuk National University Hospital, Jeonju, Republic of Korea

**Keywords:** global research equity, low- and lower-middle-income countries, lung organoids, organoids biobank, pandemic preparedness, respiratory infections, respiratory pandemic

## 1 Introduction

The next respiratory pandemic is not a question of if but when. To respond effectively, the global scientific community must be equipped with human-relevant, reproducible, and accessible models to better understand respiratory infections and evaluate potential interventions. Lung organoids (LOs)—three-dimensional, physiologically relevant models that recapitulate human airway and alveolar architecture—offer immense potential for understanding host–pathogen interactions, therapeutic responses, and vaccine development in respiratory diseases. LOs have been used to model a range of respiratory infections—for example, SARS-CoV-2 (1), influenza (2), respiratory syncytial virus (RSV) (3), and bacterial pathogens in COPD models (4). LO research has gained significant momentum over the past decade and shows a sharp rise in publications—from just one in 2011 to 929 in 2024. Nature emerged as the most cited journal, highlighting its impact in the field. The United States leads with 281 publications and over 8,000 citations (5). These trends reflect both the rapid growth and clinical relevance of LO research, emphasizing the need for continued global collaboration and investment. However, this transformative potential remains concentrated in a few high-income countries, creating a substantial gap in global research readiness. Equitable access to organoid technologies is not merely a scientific concern but a public health and preparedness imperative. Positioned as a perspective and policy statement, this manuscript integrates existing scientific knowledge with global health considerations to argue that expanding access to lung organoid technologies is essential for effective future pandemic response. In this context, we address current gaps and inequities in organoid research, propose a critical and forward-looking agenda with actionable strategies to democratize lung organoid science. We also urge policymakers and funding agencies to integrate equity-focused support for organoid research into pandemic preparedness frameworks, thereby enhancing global resilience against future respiratory pandemics.

## 2 Role of lung organoids research in respiratory pandemic

In the context of emerging respiratory threats, lung organoid systems provide a powerful platform for generating rapid functional insights across diverse genetic and demographic backgrounds. Although the derivation and expansion of new organoids generally require several weeks, the availability of established biobanks and ready-to-use organotypic model enables downstream pathogen exposure experiments to be initiated within 2–5 days (6, 7). Such accelerated functional modeling allows researchers to quickly evaluate viral tropism, transmissibility, and drug susceptibility, thereby advancing the early identification of therapeutic targets and vaccine candidates. These capabilities highlight the crucial role of organoid technologies in supporting timely and informed public health responses during the critical early stages of a pandemic. This capability is especially crucial in preparing for Disease X (8, 9)—a hypothetical but inevitable future pandemic threat. During the COVID-19 pandemic, we realized deep asymmetries in global scientific capacity (Box 1) and underscored the urgent need for scalable, adaptable technologies like organoids. In emergencies where clinical samples or field research are limited, organoids offer safe, reproducible, and ethical alternatives for studying pathogens and testing interventions. Coupled with AI modeling and omics data, they enhance predictive accuracy for emerging diseases, effectively bridging basic biology with actionable public health intelligence.

Box 1 **Lessons learned from COVID-19: unequal participation and access to human-relevant models.**

The COVID-19 pandemic highlighted a critical lesson: scientific inequity translates directly into vulnerability. While a few well-resourced countries rapidly deployed human organoid models to study SARS-CoV-2 infection, elucidate viral entry mechanisms, and screen therapeutics, most regions lacked the infrastructure, biosafety facilities, and technical expertise to conduct comparable research. For example, a study by Bipasha Bose (2021) used 3D lung organoids derived from iPSCs of diverse ethnic backgrounds to investigate variations in SARS-CoV-2 infectivity and disease severity, demonstrating how genetic and environmental factors contribute to observed disparities in COVID-19 outcomes (10). Without widespread access to technologies like organoids, the diversity of host genetics, pathogen variants, and environmental influences remains underrepresented in global datasets, limiting the comprehensiveness of biomedical responses. Similarly, Hinterberger and Stelmach examined the role of organoid technologies in global health research during the Zika and COVID-19 outbreaks (11). They emphasized that while health crises accelerated organoid research, much of the subsequent work has focused on diseases prevalent in the Global North, potentially neglecting emerging infectious diseases that disproportionately affect other regions. Ensuring equitable participation in organoid research promotes inclusivity and broadens the range of insights and innovations that benefit all populations. Thus, the uneven acceleration and subsequent deceleration of organoid research underscore the urgent need to integrate organoid technologies equitably into global health priorities.

## 3 Building organoid research capacity in low- and middle-income countries (LMICs)

Beyond accelerating research, lung organoids offer the crucial advantage of enabling personalized and population-representative studies by incorporating genetic and environmental diversity from donors worldwide (12). This inclusivity is vital to avoid repeating the inequities seen during COVID-19, where therapies and vaccines were often developed and tested in populations that did not reflect global genetic or socioeconomic diversity. The current global divide in organoid research stems from barriers like inadequate infrastructure, limited specialized training, expensive and hard-to-import reagents, and complex regulations around biobanking and cell sharing. These challenges exclude genetic and pathogen diversity from underrepresented regions, weakening global preparedness and stifling local innovation and resilience. Building organoid research capacity in low- and middle-income countries (LMICs)—which bear a disproportionate infectious disease burden—would empower scientists to produce contextually relevant data, leading to more effective and equitable health solutions. Achieving this requires confronting entrenched inequities through global strategies (Box 2) that prioritize technology transfer, capacity building, and open-access organoid biobanking backed by fair funding models.

Box 2 **Policy framework—blueprint for global organoid equity.**

We propose the following strategic recommendations for advancing global organoid research equity and enhancing respiratory pandemic preparedness.

**1. Invest in global organoids research infrastructure**

Evidence shows that lung organoid results vary significantly with culture conditions, differentiation protocols, and QC practices, underscoring how limited standardization restricts cross-study comparison (14–17). Emerging efforts such as HCA|Organoid and the Human Cell Atlas are developing shared reference datasets and benchmarks (18), but universally accepted QC standards and reporting guidelines for infection-focused lung organoid research remain absent. Inconsistent assay readouts also impede data integration during outbreaks, highlighting the need for coordinated international groups to establish consensus protocols and interoperable metadata standards. Sustainable, regionally inclusive investment is vital to expand global organoid research capacity. To close the existing gap, funding should support labs, facilities, and training in underserved regions to build local expertise and autonomy. We strongly urge international agencies like the Wellcome Trust, NIH, Gates Foundation, and WHO to commit to equitable funding that ensures organoid science benefits all.

**2. Promote open science and ethical standards**

Accelerating innovation in organoid science demands robust international collaboration grounded in openness and equity. Encouraging open-access data sharing, precompetitive collaboration, and interoperable research platforms can significantly enhance scientific progress. However, these efforts must be accompanied by thoughtful governance, particularly around the cross-border sharing of stem cell lines and patient-derived materials. Ethical biobanking, benefit-sharing, and respect for data sovereignty are essential to building trust and fairness. Global ethical frameworks must prioritize inclusivity, transparency, informed consent, and equitable benefit-sharing.

**3. Integrate organoid platforms into national preparedness plans**

Lung and airway organoid models should be integrated into national and global pandemic response plans as vital tools for rapid pathogen screening, drug testing, and vaccine evaluation. Governments must also back public-private partnerships to ensure these technologies remain scalable, affordable, and accessible during health crises.

**4. Develop rapid-response organoid biobanks and protocols**

Creating rapid-response organoid biobanks with standardized models from diverse populations is key to tackling emerging pathogens swiftly. Harmonized protocols and quality control will ensure consistency across regions. Empowering every region to grow its own lung organoids means empowering them to protect their own future. For example, large, well-established organoid biobanks—such as the Hubrecht Organoid Biobank (19)—show that patient-derived organoids can be produced, cryopreserved, and shared efficiently, allowing rapid downstream experimentation without repeated derivation. However, such facilities remain concentrated in high-income countries, limiting global access. Key gaps include the scarcity of regional biobanks in low- and middle-income settings, limited cryostorage capacity, and underrepresentation of diverse populations. Addressing these gaps will require investment in regional biobanking hubs, standardized SOPs, and equitable access and benefit-sharing frameworks.

**5. Foster interdisciplinary and cross-sector collaboration**

Future preparedness in organoid science demands interdisciplinary collaboration across biology, engineering, and data science, alongside strong partnerships between academia, industry, public health, and regulators. To scale impact globally, we must build collaborative research networks—through multinational consortia like the Human Cell Atlas—and create regional “Organoid Hubs” linked to WHO pandemic frameworks for rapid, coordinated responses to emerging health threats.

**6. Ensure sustainable funding and policy support**

Sustaining organoid research requires long-term funding and supportive policies that extend beyond emergency cycles. This includes integrating organoid science into national research agendas and pandemic financing instruments to ensure continuous readiness between outbreaks.

Establishing regional centers of excellence and promoting South–South and North–South collaborations will enable research that is contextually relevant and reduce reliance on high-income country laboratories. This distributed capacity ensures that when the next respiratory crisis arises, multiple regions—not just wealthy nations—can provide real-time data and solutions. Equally important is guiding technology development through ethical governance and inclusive participation. Transparent data-sharing, fair material transfer agreements, and community engagement must accompany biobanking and organoid derivation efforts. Only through equitable, trust-based collaboration can organoid technology serve as a bridge toward scientific solidarity rather than deepen inequalities. Integrating lung organoid research into global emergency frameworks, like the WHO Health Emergency Preparedness, Response and Resilience (HEPR) architecture (13), would formalize its role in future pandemic defense. Embedding organoid platforms within national public health institutes and academic-government partnerships can make them permanent fixtures in global surveillance and response systems.

## 4 Discussion

Global readiness for the next respiratory pandemic depends not only on scientific innovation but also on addressing the structural inequities that shape access to advanced biomedical technologies. In this policy, we expand upon several critical dimensions of disparity that currently limit the equitable development and application of lung organoid systems worldwide. First, the uneven distribution of organoid infrastructure remains a major barrier to global research equity. High-income countries continue to dominate access to specialized facilities, high-cost equipment, and advanced biobanking capabilities, while many low- and middle-income regions lack even the foundational resources required to initiate organoid programs. These gaps restrict the timely generation of region-specific biological data that would otherwise strengthen global pandemic preparedness. Second, significant technical challenges in standardizing organoid protocols hinder meaningful collaboration and data comparability across international research groups. Differences in culture conditions, quality control metrics, and analytical pipelines lead to variable outcomes, making it difficult to establish shared reference models. Addressing these technical inconsistencies is essential for creating a harmonized global organoid research ecosystem. Third, there is a persistent shortage of a trained workforce with expertise in stem cell biology, organoid engineering, and advanced imaging and analytical platforms. Many regions lack access to sustained training pipelines, resulting in a widening skills gap that slows both scientific progress and technology adoption. Developing coordinated, internationally supported training initiatives will be critical for expanding global capacity.

Given the existing limitations in infrastructure, workforce capacity, and equitable access, integrating organoid platforms directly into national preparedness plans may indeed be premature at this stage. To address this, we frame such integration as a gradual, context-dependent process rather than an immediate policy action. In this framework, we emphasize organoid technologies as a promising adjunct to existing public health and laboratory systems. We advocate that their effective incorporation into preparedness strategies will require sustained investment, coordinate capacity-building efforts, and strengthen international collaboration to overcome the disparities in organoid research.

Furthermore, funding and governance limitations continue to constrain sustainable organoid research growth in resource-limited settings. Short funding cycles, fragmented investment strategies, and limited institutional support weaken long-term infrastructure planning and impede the creation of resilient research networks. Strengthening financial and governance frameworks is therefore essential for supporting durable organoid innovation. Finally, the ethical and regulatory complexities of cross-border organoid sharing, an area that requires urgent attention. Divergent national regulations, concerns about data sovereignty, and inconsistent ethical oversight can delay or prevent the exchange of biological materials. Harmonized policies are needed to ensure that organoid resources can be shared responsibly, equitably, and efficiently during global health emergencies. Together, these provide a clearer policy roadmap for democratizing lung organoid science.

## 5 Conclusion

The promise of organoid models goes beyond scientific advancement—it offers a chance to reshape global health equity. Democratizing access to this technology will boost our collective preparedness and ensure that the fruits of discovery are shared by all. With “Disease X” an ever-present threat, investing in equitable organoid infrastructure today could determine how effectively humanity responds tomorrow. Strengthening organoid research equity is not a luxury for low- and middle-income countries—it is essential for global health security in the twenty-first century. Lung organoids can evolve from a scientific breakthrough into a cornerstone of worldwide resilience in respiratory pandemic. Through coordinated efforts to close infrastructure disparities, establish robust standardization practices, develop a skilled workforce, ensure sustainable funding, and remove regulatory obstacles, the international community can create a more inclusive and strategically prepared research landscape. Such a strengthened system will be far better positioned to generate timely insights and support an effective response when the next respiratory pandemic emerges.
